# Characteristics and outcomes in patients with atrial fibrillation receiving direct oral anticoagulants in off-label doses

**DOI:** 10.1186/s12872-020-01340-4

**Published:** 2020-02-03

**Authors:** Alexandros Briasoulis, Yubo Gao, Chakradhari Inampudi, Paulino Alvarez, Rabea Asleh, Elizabeth Chrischilles, Enrique C. Leira, Mary Vaughan-Sarrazin

**Affiliations:** 1 Division of Cardiovascular Diseases, Section of Heart Failure and Transplant, University of Iowa College of Medicine, Iowa City, IA USA; 2 Division of Cardiovascular Medicine, 200 Hawkins Dr, Iowa City, IA 52242 USA; 3 Division of General Medicine, University of Iowa College of Medicine, Iowa City, IA USA; 4 Department of Epidemiology, University of Iowa College of Public Health, Iowa City, IA USA; 5 Departments of Neurology and Neurosurgery, University of Iowa College of Medicine, Iowa City, USA; 6 Comprehensive Access and Delivery Research and Evaluation Center, Iowa City VA Medical Center, IA, 200 Hawkins Dr, Iowa City, IA 52242 USA

**Keywords:** Atrial fibrillation, Stroke, Bleeding, Direct oral anticoagulants

## Abstract

**Background:**

We evaluated adherence to dosing criteria for patients with atrial fibrillation (AF) taking dabigatran or rivaroxaban and the impact of off-label dosing on thromboembolic and bleeding risk.

**Methods:**

We used data for a retrospective cohort from a large U.S. health plan for Medicare beneficiaries age > =65 years with AF who initiated dabigatran or rivaroxaban during 2010–2016. Stroke and major bleeding were quantified in patients who were eligible for low dose but received standard dose, and in patients who were eligible for standard dose but received low dose.

**Results:**

We identified 8035 and 19,712 patients who initiated dabigatran or rivaroxaban, respectively. Overall, 1401 (17.4%) and 7820 (39.7%) patients who received dabigatran and rivaroxaban met criteria for low dose, respectively. Of those, 959 (68.5%) and 3904 (49.9%) received standard dose. In contrast, 1013 (15.3%) and 2551 (21.5%) of patients eligible for standard dose dabigatran and rivaroxaban received low dose**.** Mean follow-up for patients eligible for low and standard dose dabigatran and rivaroxaban were 13.9, 15.1, 10.1, and 12.3 months, respectively. In unadjusted analyses, patients eligible for low or standard dose dabigatran and rivaroxaban but receiving off-label dose, had no differences in the rates of ischemic stroke. Among patients who met criteria for standard dose direct oral anticoagulants (DOAC), use of low dose was associated with significantly higher risk of any major bleeding (Dabigatran: HR = 1.44; 95% CI 1.14–1.8, *P* = 0.002, Rivaroxaban HR 1.34, 95% CI 1.11–1.6, *P* = 0.002) and gastrointestinal bleeding (Dabigatran: HR = 1.48; 95% CI 1.08–2, *P* = 0.016). In patients who met criteria for low dose DOACs, there was lower risk of major bleeding (Dabigatran: HR = 0.59; 95% CI 0.43–0.8, *P* < 0.001), gastrointestinal (Rivaroxaban: HR 0.79; 95% CI 0.64–0.98, *P* = 0.03) and intracranial bleeding (Dabigatran: HR = 0.33; 95% CI 0.12–0.9, *P* = 0.001) with standard dosing. After propensity matching, use of off-label doses was not associated with stroke, major, gastrointestinal or intracranial bleeding for either dabigatran or rivaroxaban.

**Conclusions:**

While a significant number of patients receive higher or lower dose of dabigatran and rivaroxaban than recommended, we found no evidence of significant impact on thromboembolic or hemorrhagic outcomes.

## Background

Patients with Atrial fibrillation (AF) have a higher risk for stroke or systemic embolism, death and disability [1]. Oral anticoagulants, either vitamin K antagonist (VKA) or direct oral anticoagulants (DOACs) reduce that thromboembolic risk by about two-thirds irrespective of baseline risk [2]. However, the use of anticoagulation is associated with increased risk of bleeding, with intracranial hemorrhage (ICH) being the most serious bleeding complication [3]. Randomized controlled trials (RCT) of DOACs [Dabigatran, Rivaroxaban, Apixaban, and Edoxaban] have demonstrated similar protection against ischemic stroke but lower rates of ICH compared with VKAs [4–7].

The RCTs of DOACs in AF patients used dose adjustments based on patient characteristics such age, weight, renal function and the use of concomitant medications. A reduced dose of 75 mg twice daily of dabigatran is recommended to decrease bleeding risk in patients with creatinine clearance (CrCl) 15–30 mL/minute, or co- administration of a strong P-glycoprotein [P-gp] inhibitor (e.g., dronedarone) in patients with CrCl 30–50 mL/minute [8, 9]. With regards to rivaroxaban, a dose reduction to 15 mg daily is recommended in patients with CrCl 15–50 mL/minute, and concomitant use of a dual P-gp and cytochrome-3A4 [P-gp-Cyp3A4] inhibitor should be avoided to prevent potential increased rivaroxaban concentration [10]. Administration of lower apixaban dose 2.5 mg twice daily is indicated if 2 of the following 3 criteria are met: age > 80 years, weight < than 60 kg, and serum Creatinine > 1.5 mg/dl [11].

Since the use of DOACs became widespread, deviations from the recommended dosing are not infrequent [12, 13]. Analysis of 5738 patients treated with DOACs from the ORBITA-AF II registry showed that 9.4% of patients were under-dosed and 3.4% were overdosed. Overdosing was associated with significantly increased all-cause mortality whereas under-dosing was associated with increased cardiovascular hospitalizations [14]. A subsequent analysis of 7925 AF patients treated with DOACs from the same registry showed that 16% of patients were on reduced doses with many of these doses adjustments (57%) not following the recommended doses [15]. Nevertheless, after risk- adjustment, the use of lower-than-recommended dose resulted in similar thromboembolic and bleeding risk compared to appropriately dosed DOAC use [15].

We hypothesized that a sizeable number of DOAC prescriptions do not adhere to the Food and Drug Administration (FDA) dosing criteria and may increase thromboembolic and bleeding events. The purpose of our study was to: 1) examine characteristics and predictors of low dose use among patients who meet FDA criteria for standard dose, or standard dose use among patients who meet FDA criteria for low dose, among patients who initiate dabigatran and rivaroxaban, ii) compare the risk of ischemic stroke and bleeding events in patients receiving off-label low dose or off-label standard dose to patients receiving FDA-recommended doses in a community-based sample of elderly Medicare beneficiaries with AF enrolled in a large U.S. health plan.

## Methods

### Data source

We designed a new user retrospective cohort study using data for Medicare beneficiaries enrolled in a large U.S. health plan with prescription drug coverage. Medical (inpatient visit, outpatient physician visits) and pharmacy claims with detailed prescription fill information from October 1, 2010 through December 31, 2016 were analyzed. In addition, the data also includes laboratory test results (such as serum creatinine) for Medicare beneficiaries enrolled in managed care plans. The study was non-human subject research by the University of Iowa institutional review board because it involved analysis of an existing database that was fully de-identified.

### Patient population

We analyzed claims for Medicare beneficiaries age > = 65 years with newly diagnosed atrial fibrillation (AF) between 2010 and 2016 (dabigatran was approved by FDA in October 2010 followed by rivaroxaban approval in November 2011). We identified patients who initiated dabigatran 150 mg twice daily (standard dose) or 75 mg twice daily (low dose), or rivaroxaban 20 mg daily (standard dose) or 15 mg daily. We did not include patients receiving apixaban as information to assess dosing criteria such as patient weight was not available in our data, and we did not include patients receiving edoxaban due to relatively low use of this drug during our time frame. Patients were categorized into mutually exclusive groups according to the first DOAC and DOAC dose received.

Patients were excluded if they did not have a diagnosis of AF during the 12 months prior to initiating the DOAC, where AF was defined as International Classification of Diseases–Ninth Revision–Clinical Modification [ICD-9-CM] code 427.31 or ICD-Tenth Revision [ICD-10] code I48.0, I48.1, I48.2, I48.3, I48.4, or I48.91, as primary or secondary diagnosis. Additionally, we excluded patients who: i) were younger than 65 years at the time of diagnosis, ii) had a diagnosis indicating pulmonary embolism or deep vein thrombosis within 8 weeks prior to initiating the DOAC, iii) underwent hip surgery within 6 weeks prior to initiating the DOAC, or iv) were not enrolled in the health care plan for at least 1 year prior to initiating the DOAC.

Patients who initiated dabigatran or rivaroxaban were categorized to approximate dosing criteria, using the most recent estimated glomerular filtration rate (eGFR) available prior to initiating the DOAC as a proxy for creatinine clearance. eGFR was calculated based on Modification of Diet in Renal Disease (MDRD) Study equation or Chronic Kidney Disease Epidemiology Collaboration (CKD-EPI) equation [16, 17]. Patients who received dabigatran were deemed to meet criteria for low dose if they had severe renal disease (defined as eGFR < 30 mL/minute/ 1.73 m2) or had moderate renal disease and concurrent use of a p-gp inhibitor (where moderate renal disease was defined as eGFR 30–50 mL/minute/1.73 m2 and p-gp inhibitors included dronedarone, cyclosporine, itraconazole, tacrolimus, ketoconazole). Patients who received rivaroxaban were deemed to meet criteria for low dose if they had eGFR< 50 or concomitant use of a dual P-gp-Cyp3A4 inhibitor (including ketoconazole, fluconazole, itraconazole, cobicistat, conivaptan, indinavir, voriconazole, posaconazole, nefazodone HCL, ritonavir, saquinavir, telithromycin). Patients with no valid GFR for assessing renal function were excluded. Among patients eligible for low doses of dabigatran or rivaroxaban, 87.4 and 86.5% had available eGFR whereas among those eligible for standard dose dabigatran or rivaroxaban, 54.9 and 56% had available eGFR respectively. We performed sensitivity analysis for DOAC dose adjustments based on eGFR only, excluding drug interactions.

### Covariates

Data on patient-level characteristics such as demographics, comorbid conditions, concurrent medication use, and prior health services utilization were extracted from health care plan enrollment data and inpatient, outpatient, and physician claims. Comorbid diseases were identified by ICD-9-CM/ICD-10 diagnoses on claims during the 12 months preceding the date of first DOAC fill. We used the Charlson Comorbidity Index to estimate patients’ overall comorbidity status [18]. We also identified all conditions in the CHA2DS2-VASc stroke risk score (congestive heart failure diagnosis, female sex, hypertension diagnosis, diabetes, age, prior stroke or transient ischemic attack, and vascular disease diagnosis). History of major bleeding was defined as any prior major bleeding, gastrointestinal bleeding, intracranial hemorrhage, or prior receipt of transfusion. Additional conditions included liver disease, alcohol abuse, obesity, chronic obstructive pulmonary disease, peripheral vascular disease, pulmonary circulation disease, heart valve disease, history of coronary revascularization, history of implantable cardiac device, and prior pulmonary embolism or deep vein thrombosis. We also extracted data on medication use at the time of DOAC initiation (p-glycoprotein inhibitors, cytochrome 3A4 inhibitors, insulin, statins, beta blockers, angiotensin-converting-enzyme (ACE) inhibitors, angiotensin-receptor blockers (ARBs), calcium channel blockers, prescription antiplatelets (e.g., clopidogrel), proton pump inhibitors and non-steroidal anti-inflammatory drugs. (A list of included drugs and ICD9/ICD10 codes for comorbid conditions is provided in Additional file 1: Table S1). Medications were considered concomitant if the patient had supply within 90 days from the DOAC prescription. We also identified patients with a history of warfarin use prior to initiating the DOAC.

### Endpoints

We selected the following clinical endpoints: i) ischemic stroke, ii) any major bleeding, iii) gastrointestinal bleeding (GI), iv) intracranial bleeding based on the primary ICD-9-CM/ICD-10 diagnosis on inpatient claims for acute care stays (definitions provided in Additional file 1: Table S1). We also examined drug discontinuation, as defined by the date of last fill for the original DOAC and dose. Patients were followed from the date of the initial DOAC prescription until December 31, 2016 or lapse of health plan enrollment (due to death or other reason), or cessation of the initial DOAC dose.

### Statistical analysis

Analyses were conducted separately for patients who initiated dabigatran and rivaroxaban, and by dose eligibility. For example, among patients who received dabigatran and met criteria for low dose dabigatran, we compared characteristics and outcomes among patients who received standard dose in contrast to dosing criteria vs. those who received the recommended low dose. Similarly, among patients who received dabigatran and met criteria for standard dose, we compared patients who received low dose vs. those who received the recommended standard dose. We compared demographic characteristics, comorbid diseases, and concurrent medication use among patients on different doses using the chi-square test.

We examined rates of ischemic stroke, any major bleeding, GI hemorrhage, and intracranial hemorrhage per patient-year of follow-up in the full sample and in propensity matched patients. Specifically, we performed 2-way nearest-neighbor propensity-matching to create groups of patients receiving low dose or standard dose that were balanced with respect to observed patient characteristics. Propensity matching was conducted separately for patients qualifying for low or standard dose, and for patients on dabigatran or rivaroxaban (i.e., four separate propensity matched samples). We assessed covariate balance in propensity-matched samples using standardized differences between patients receiving low or standard dose, where differences less than 10% indicate satisfactory balance. Because standardized differences remained greater than 10% for a small number of covariates, we further adjusted for unbalanced covariates if they were related to the outcome of interest using Cox proportional hazards regression models. We then calculated rates of each endpoint per patient year of follow-up in unmatched and propensity-matched samples. Statistical significance was assessed using Cox proportional hazards regression models that censored for end of follow-up (December 31, 2016), medication cessation, or disenrollment from the managed care plan for any reason. Results were reported as hazard ratios (HR) with 95% confidence intervals (CI) for each drug and recommended dose, where the HR reflects the outcome rate for off-label dosing relative to FDA-recommended dose. Finally, one sensitivity analysis was performed in which drug dosing criteria was based on GFR measurements only, without considering pharmacologic interactions reflected in FDA criteria.

All analyses were conducted using SAS with 2-tailed level of significance set at 0.05.

## Results

### Patient characteristic

We identified 8035 patients with valid GFR measurements including 6580 on standard dabigatran dose and 1455 patients on low dose (18.1%); 19,712 patients were rivaroxaban including 13,245 on standard dose rivaroxaban and 6467 on low dose (32.8%). We identified significant differences in baseline characteristics between different does of dabigatran and rivaroxaban before propensity matching (Tables 1 and 2). Among dabigatran patients, those taking low dose were more likely to be over 75 years (*P* < 0.001), female (*P* = 0.01), have CKD III or more advanced (*P* < 0.001) and higher burden of comorbidities as indicated by higher Charlson comorbidity index (low dose: 5.52 ± 3.75 vs. high dose: 4.17 ± 3.47, *P* < 0.001). Among rivaroxaban patients, low dose was more frequently prescribed to patients over 75 years (*P* < 0.001), females (*P* = 0.03), patients with CKD III or more advanced (*P* < 0.001) and patients with higher comorbidity burden (low dose: 5.05 ± 3.65 vs. high dose: 3.71 ± 3.34, *P* < 0.001).

**Table 1:** Characteristics of patients taking standard (150 mg) or reduced (75 mg) dose dabigatran

	Reduced dose (75 mg twice daily)	Standard Dose (150 mg twice daily)	*P*-value
Total number of patients	1455	6580	
Age Category, years			*< 0.001*
65–69	7.1%	18.0%	
70 to 74	13.7%	26.8%	
75 to 79	18.8%	25.4%	
80 to 84	26.0%	18.1%	
85 to 89	16.8%	7.1%	
90 or over	17.5%	4.7%	
Sex			*< 0.001*
Female	56.3%	47.9%	
Male	43.7%	52.1%	
Race Category			*< 0.001*
White	84.1%	87.5%	
Black	10.2%	6.9%	
Hispanic	2.5%	1.7%	
Asian	1.4%	0.6%	
Other	1.9%	3.2%	
Smoking	14.0%	16.7%	0.01
Alcohol use	2.0%	2.3%	< 0.001
Weight Category (based on ICD-9 and ICD-10 codes for BMI Category)			*< 0.001*
Under-Weight	1.2%	0.6%	
Healthy or Overweight	10.1%	8.0%	
Obese or Severe Obese	20.1%	23.9%	
Not available	68.5%	67.5%	
Comorbid Conditions			
Prior Stroke	26.8%	24.4%	0.06
Prior major bleeding from Diagnosis	26.7%	24.5%	0.07
Gastrointestinal bleeding	0	13.0%	0.24
Cerebral bleeding	0	0.8%	0.7
Diabetes	50.7%	48.9%	0.2
Prior AMI	8.0%	5.2%	< 0.001
Liver Disease	3.4%	2.6%	0.13
Heart Failure	48.6%	31.3%	< 0.001
Hypertension	96.0%	93.4%	< 0.001
Ischemic cardiomyopathy	55.5%	48.0%	< 0.001
Pulmonary	16.6%	11.4%	< 0.001
COPD	36.4%	31.8%	< 0.001
Transfusion from Procedure	3.8%	2.7%	0.02
Revascularization	17.3%	15.0%	0.03
Implantable Devices	16.9%	13.8%	0.002
Valve Disease	44.7%	40.3%	0.002
Renal Disease (ICD-9 and ICD-10 codes)			< 0.001
None or Mild (Stage I, II)	53.4%	81.7%	
Moderate (Stage III)	36.0%	16.5%	
Severe (Stage IV, V)	10.6%	1.7%	
Concurrent Drugs (+/− 90 days of initiating DOAC)			
SSRI/SNRI	33.5%	31.0%	0.05
Strong and moderate P-GP inhibitors	21.4%	22.7%	0.28
P-GP inducers	16.4%	18.8%	0.03
Strong P-GP and CYP3A4 dual inhibitors	22.7%	22.5%	0.8
P-GP and CYP3A4 inducers	16.4%	18.8%	0.03
ACE inhibitors	70.1%	68.0%	0.12
Warfarin	20.8%	25.0%	< 0.001
Angiotensin receptor blockers	41.0%	39.3%	0.2
Beta blockers	92.1%	89.1%	< 0.001
Calcium channel blockers	66.8%	63.8%	0.03
Digoxin	31.1%	29.4%	0.2
Proton pump inhibitors	60.5%	56.4%	0.004
NSAIDs	49.6%	51.2%	0.29
Antiplatelets	32.0%	26.5%	< 0.001
Insulin	17.0%	12.9%	< 0.001
Statins	79.6%	79.4%	0.86
Antiarrhythmics	47.3%	48.0%	0.6
Estimated Glomerular Filtration rate (ml/min/1.73 m2)			*< 0.001*
< 30	12.3%	2.5%	
30–60	62.2%	45.8%	
60–90	23.8%	46.0%	
> = 90	1.8%	5.7%	
CCI(Charlson Comorbodity Index), mean (standard deviation)	5.52 (3.75)	4.17(3.47)	< 0.001

Abbreviations: *ACE* Angiotensin converting enzyme, *AMI* Acute myocardial infarction, *COPD* Chronic obstructive pulmonary disease, *ICD* International classification of diseases, *NSAIDS* Non-steroidal antiinflammatory drugs, *P-GP* P-glycoprotein, *CYP* Cytochrome

**Table 2:** Characteristics of patients taking standard (20 mg) or reduced (15 mg) dose rivaroxaban

	Reduced Dose (15 mg daily)	Standard Dose (20 mg daily)	*P*-value
Total number of patients	6467	13,245	
Age Category, years			*< 0.001*
65–69	7.9%	18.2%	
70 to 74	14.8%	29.0%	
75 to 79	20.7%	25.6%	
80 to 84	25.4%	17.0%	
85 to 89	19.8%	7.8%	
90 or over	11.4%	2.4%	
Sex			*< 0.001*
Female	57.1%	46.2%	
Male	42.9%	53.8%	
Race Category			*< 0.001*
White	85.2%	85.8%	
Black	9.0%	8.0%	
Hispanic	2.2%	1.6%	
Asian	0.8%	0.8%	
Other	2.8%	3.8%	
Smoking	20.2%	22.8%	< 0.001
Alcohol	2.3%	3.2%	< 0.001
Weight Category (based on ICD-9 and ICD-10 codes for BMI Category)			*< 0.001*
Under-Weight	2.3%	1.3%	
Healthy or Overweight	19.1%	14.3%	
Obese or Severe Obese	26.2%	31.2%	
Not available	52.4%	53.2%	
Comorbid Conditions			
Prior Stroke	28.2%	22.4%	< 0.001
Prior major bleeding from Diagnosis	26.6%	23.9%	< 0.001
Gastrointestinal bleeding	15.2%	13.5%	0.002
Cerebral bleeding	1.3%	0.8%	< 0.001
Diabetes	51.0%	47.7%	< 0.001
Prior AMI	9.0%	5.7%	< 0.001
Liver Disease	2.9%	3.1%	0.35
Heart Failure	35.6%	24.3%	< 0.001
Hypertension	94.6%	92.1%	< 0.001
Ischemic cardiomyopathy	47.7%	40.2%	< 0.001
Pulmonary	15.9%	11.1%	< 0.001
COPD	36.3%	31.4%	< 0.001
Transfusion from Procedure	5.0%	2.3%	< 0.001
Revascularization	16.5%	13.7%	< 0.001
Implantable Devices	16.7%	12.5%	< 0.001
Valve Disease	40.6%	36.4%	0.002
Renal Disease (ICD-9 and ICD-10 codes)			< 0.001
None or Mild (Stage I, II)	54.8%	83.9%	
Moderate (Stage III)	37.5%	14.5%	
Severe (Stage IV, V)	7.7%	1.5%	
Concurrent Drugs (+/− 90 days of initiating DOAC)			
SSRI/SNRI	33.7%	29.1%	< 0.001
Strong and moderate P-GP inhibitors	18.3%	18.5%	0.63
P-GP inducers	18.5%	16.5%	< 0.001
Strong P-GP and CYP3A4 dual inhibitors	22.7%	20.8%	0.002
P-GP and CYP3A4 inducers	18.5%	16.5%	< 0.001
ACE inhibitors	67.4%	64.3%	< 0.001
Warfarin	15.0%	16.1%	0.045
Angiotensin receptor blockers	40.0%	36.7%	< 0.001
Beta blockers	88.1%	86.5%	0.015
Calcium channel blockers	64.5%	59.4%	< 0.001
Digoxin	23.1%	20.8%	< 0.001
Proton pump inhibitors	58.3%	52.3%	< 0.001
NSAIDs	52.9%	50.1%	< 0.001
Antiplatelets	30.2%	24.3%	< 0.001
Insulin	14.3%	10.8%	< 0.001
Statins	79.3%	77.6%	0.006
Antiarrhythmics	40.4%	43.1%	< 0.001
Estimated Glomerular Filtration rate (ml/min/1.73 m2)			< 0.001
< 30	7.5%	1.3%	
30–60	61.1%	35.2%	
60–90	28.8%	56.7%	
> =90	2.6%	6.8%	
CCI(Charlson Comorbodity Index), mean (standard deviation)	5.05(3.65)	3.71(3.34)	< 0.001

Abbreviations: *ACE* Angiotensin converting enzyme, *AMI* Acute myocardial infarction, *COPD* Chronic obstructive pulmonary disease, *ICD* International classification of diseases, *NSAIDS* Non-steroidal antiinflammatory drugs, *P-GP* P-glycoprotein, *CYP* Cytochrome

As shown on Tables 3, 4, 5 and 6, 1401 (17.4%) and 7820 (39.7%) patients who received dabigatran and rivaroxaban met criteria for low dose, respectively. Of those, 959 (68.5%) and 3904 (49.9%) received standard dose of dabigatran and rivaroxaban respectively. In contrast, 1013 (15.3%) and 2551 (21.5%) of patients eligible for standard dose dabigatran and rivaroxaban received low dose**.** Patients older than 75 years, females, African Americans, and patients with history of major bleeding or heart failure were more likely to receive lower than recommended dose of dabigatran or rivaroxaban (Tables 3, 4, 5 and 6). Conversely, patients eligible for low dose dabigatran or rivaroxaban that received standard dose were more likely younger with lower rates of advanced CKD.

**Table 3:** Bivariable associations between low dose dabigatran eligible patients’ characteristics on low or standard dose of dabigatran

	Low dose eligible that received low dose Dabigatran (*n* = 442)	Low dose eligible that received standard dose Dabigatran (*n* = 959)	*P* value	Before Matching Standardized Difference	After Matching (*N* = 366 vs 366) Standardized Difference
Year			0.001		
2010–2012	57.7%	69.3%		0.244	0.228
2013	16.1%	10.3%		0.170	0.289
2014	10.9%	8.1%		0.093	0.047
2015	7.7%	6.5%		0.048	0
2016–2017	7.7%	5.7%		0.078	0.011
Region			0.1		
Midwest	15.4%	12.4%		0.086	0.008
Northeast	0.5%	1.4%		0.096	0.141
South	73.3%	76.7%		0.080	0
West	10.9%	9.5%		0.045	0.035
Age Category			< 0.001		
65–69	7.9%	14.0%		0.195	0
70 to 74	16.5%	24.2%		0.192	0
75 to 79	18.6%	28.5%		0.235	0
80 to 84	25.1%	19.3%		0.140	0
85 to 89	13.1%	7.8%		0.174	0
90 or over	18.8%	6.3%		0.385	0
Sex			0.2		
Female	57.9%	54.3%		0.072	0
Male	42.1%	45.7%			
Race Category					
White	84.2%	85.0%		0.023	0
Black	10.4%	8.9%		0.052	0
Hispanic	2.5%	2.4%		0.006	0
Other					0
Smoking			0.04		
Yes	11.5%	15.7%		0.123	0.055
No	88.5%	84.3%			
Weight Category (based on ICD-9 and ICD-10 codes for BMI Category)			0.02		
Underweight	1.6%	0.6%		0.092	0
Healthy or Overweight	8.6%	6.3%		0.089	0.062
Obese or Severe Obese	20.4%	26.3%		0.140	0.013
Others	69.5%	66.8%		0.056	0.024
Comorbid Conditions					
Prior Stroke	26.7%	27.4%	0.8	0.016	0.042
Prior major bleeding	29.0%	28.2%	0.8	0.018	0.006
Diabetes	55.0%	54.0%	0.7	0.019	0.077
Prior AMI	10.6%	6.2%	0.003	0.162	0.147
Liver Disease	2.9%	3.2%	0.8	0.017	0
Heart Failure	56.8%	40.7%	< 0.001	0.327	0.265
Hypertension	98.2%	96.1%	0.04	0.124	0.082
Ischemic cardiomyopathy	60.4%	57.2%	0.3	0.064	0.089
Pulmonary	18.8%	13.3%	0.008	0.148	0.066
COPD	37.8%	36.2%	0.6	0.033	0.034
Transfusion from Procedure	5.4%	4.3%	0.3	0.054	0.051
Revascularization	18.6%	17.8%	0.7	0.019	0.043
Implantable Devices	20.1%	17.5%	0.2	0.067	0
Valve Disease	45.9%	42.0%	0.2	0.079	0.099
Concurrent Drugs (+/− 90 days of initiating DOAC)					
SSRI/SNRI	32.6%	36.5%	0.15	0.083	0.023
Strong and moderate p-gp inhibitors	53.5%	79.9%	< 0.001	0.580	0.068
Warfarin	22.6%	31.0%	0.001	0.189	0.153
Strong p-gp and cyp3a4 dual inhibitors	35.7%	47.5%	< 0.001	0.241	0.089
ACE inhibitors	74.0%	72.7%	0.6	0.029	0.044
Angiotensin receptor blockers	46.6%	45.9%	0.8	0.015	0.066
Beta blockers	95.2%	92.9%	0.09	0.099	0.165
Calcium channel blockers	69.7%	67.5%	0.4	0.048	0.098
Digoxin	28.7%	33.8%	0.06	0.109	0.083
Proton pump inhibitors	64.7%	63.2%	0.6	0.032	0.119
NSAIDs	52.7%	55.4%	0.4	0.053	0.055
Antiplatelets	35.3%	31.3%	0.13	0.085	0.099
Insulin	22.9%	17.6%	0.02	0.130	0.206
Statins	80.8%	83.3%	0.24	0.066	0.007
Renal Disease					
Moderate (GFR 30–60 ml/min/1.73 m2)	45.7%	74.9%	< 0.001	0.6245	0.0439
Severe (GFR < 30 ml/min/1.73 m2)	54.3%	25.1%			

Abbreviations: *ACE* Angiotensin converting enzyme, *AMI* Acute myocardial infarction, *COPD* Chronic obstructive pulmonary disease, *ICD* International classification of diseases, *NSAIDS* Non-steroidal antiinflammatory drugs, *P-GP* P-glycoprotein, *CYP* Cytochrome

**Table 4:** Bivariable associations between standard dose dabigatran eligible patients’ characteristics on low or standard dose of dabigatran

	Standard dose eligible that received low dose Dabigatran (*n* = 1013)	Standard dose eligible that received standard dose Dabigatran (*n* = 5621)	*P* value	Before Matching Standardized Difference	After Matching (*N* = 1001 vs 1001) Standardized Difference
Year			< 0.001		
2010–12	50.0%	55.7%		0.116	0.198
2013	18.6%	13.6%		0.137	0.174
2014	11.6%	11.4%		0.008	0.032
2015	10.9%	10.1%		0.026	0.047
2016–17	9.0%	9.3%		0.009	0.040
Region			0.8		
Midwest	12.3%	12.9%		0.017	0.024
Northeast	1.7%	1.5%		0.018	0.016
South	73.7%	74.3%		0.012	0.009
West	12.2%	11.4%		0.027	0.006
Age Category			< 0.001		
65–69	6.7%	18.7%		0.365	0
70 to 74	12.5%	27.3%		0.3755	0
75 to 79	19.0%	24.9%		0.143	0
80 to 84	26.4%	17.9%		0.206	0
85 to 89	18.5%	6.9%		0.351	0
90 or over	17.0%	4.4%		0.415	0
Sex			< 0.001		
Female	55.6%	46.8%		0.176	0
Male	44.4%	53.2%			
Race Category			< 0.001		
White	84.0%	87.9%		0.113	0
Black	10.1%	6.6%		0.126	0
Hispanic	2.5%	1.6%		0.059	0
Asian	1.2%	0.6%		0.066	0
Other	2.3%	3.3%		0.060	0
Smoker	15.0%	16.8%	0.15	0.049	0.037
Weight Category (based on ICD-9/ ICD-10 codes for BMI)			0.003		
Under Weight	1.1%	0.6%		0.057	0.031
Healthy or Overweight	10.8%	8.3%		0.082	0.013
Obese or Severe Obese	20.0%	23.4%		0.084	0.054
Others	68.1%	67.6%		0.010	0.061
Comorbid Conditions					
Prior Stroke	26.9%	23.9%	0.04	0.067	0.011
Prior Major Bleeding	25.8%	23.9%	0.2	0.044	0.042
Diabetes	48.9%	48.0%	0.6	0.018	0.066
Prior AMI	6.9%	5.0%	0.01	0.082	0.103
Liver Disease	3.6%	2.5%	0.07	0.059	0.126
Heart Failure	45.0%	29.7%	< 0.001	0.320	0.274
Hypertension	95.1%	93.0%	0.01	0.089	0.088
Ischemic Cardiomyopathy	53.4%	46.4%	< 0.001	0.140	0.110
Pulmonary Circulatory Disease	15.6%	11.0%	< 0.001	0.135	0.083
COPD	35.7%	31.0%	0.003	0.1	0.115
Blood Transfusion	3.2%	2.4%	0.18	0.044	0
Revascularization	16.8%	14.5%	0.05	0.063	0.075
Implantable cardiac device	15.5%	13.1%	0.04	0.068	0.011
Valve Disease	44.1%	40.0%	0.015	0.083	0.024
Concurrent Drugs (+/− 90 days of initiating DOAC)					
SSRI/SNRI	34.0%	30.0%	0.01	0.085	0.126
Strong and moderate p-gp inhibitors	7.3%	12.9%	< 0.001	0.188	0.135
Warfarin	19.9%	24.0%	0.004	0.099	0.237
Strong p-gp and cyp3a4 dual inhibitors	17.0%	18.2%	0.3	0.0325	0.032
ACE inhibitors	68.4%	67.2%	0.45	0.0257	0.081
Angiotensin receptor blockers	38.6%	38.1%	0.8	0.0094	0.010
Beta blockers	90.7%	88.4%	0.03	0.0759	0.108
Calcium channel blockers	65.5%	63.2%	0.15	0.0488	0.040
Digoxin	32.1%	28.7%	0.03	0.0741	0.002
Proton pump inhibitors	58.7%	55.2%	0.04	0.0707	0.008
NSAIDs	48.3%	50.4%	0.2	0.0433	0.04
Antiplatelets	30.5%	25.7%	0.0015	0.1064	0.093
Insulin	14.5%	12.1%	0.03	0.072	0.137
Statins	79.1%	78.7%	0.8	0.009	0.079
Renal Disease			< 0.001		
None or Mild	33.6%	57.2%		0.489	0.255
Moderate (GFR 30–60 ml/min/1.73 m2)	66.4%	42.8%			
Severe (GFR < 30 ml/min/1.73 m2)	0.0%	0.0%			

Abbreviations: *ACE* Angiotensin converting enzyme, *AMI* Acute myocardial infarction, *COPD* Chronic obstructive pulmonary disease, *ICD* International classification of diseases, *NSAIDS* Non-steroidal antiinflammatory drugs, *P-GP* P-glycoprotein, *CYP* Cytochrome

**Table 5:** Bivariable associations between low dose rivaroxaban eligible patients’ characteristics on low or standard dose of rivaroxaban

	Low dose eligible that received low dose Rivaroxaban (*n* = 3916)	Low dose eligible that received standard dose Rivaroxaban (*n* = 3904)	*P* value	Before Matching Standardized Difference	After Matching (*N* = 2703 vs 2703) Standardized Difference
Year			0.2		
2010–2012	7.2%	6.7%		0.021	0.028
2013	18.3%	17.6%		0.017	0.0308
2014	23.0%	23.0%		0.002	0.016
2015	22.9%	21.8%		0.024	0.003
2016–2017	28.6%	30.9%		0.050	0.058
Region			0.04		
Midwest	15.2%	13.0%		0.064	0.084
Northeast	1.2%	1.1%		0.009	0.016
South	73.0%	75.0%		0.044	0.028
West	10.5%	10.9%		0.013	0.061
Age Category			< 0.001		
65–69	7.5%	14.5%		0.225	0
70 to 74	14.0%	26.0%		0.302	0
75 to 79	20.1%	25.8%		0.135	0
80 to 84	25.5%	19.7%		0.139	0
85 to 89	20.5%	10.1%		0.291	0
90 or over	12.4%	3.9%		0.311	0
Sex			0.0008		
Female	56.0%	52.3%		0.076	0
Male	44.0%	47.7%			
Race Category			0.7		
White	84.5%	83.4%		0.031	0
Black	10.2%	10.7%		0.017	0
Hispanic	2.0%	2.2%		0.011	0
Asian	0.7%	0.8%		0.015	0
Other	2.7%	3.0%		0.019	0
Smoker	19.6%	22.5%	0.001	0.073	0.033
Weight Category (ICD9/ICD-10 codes for BMI)			< 0.0001		
Under Weight	1.9%	1.2%		0.053	0.025
Healthy or Overweight	18.4%	16.1%		0.062	0.008
Obese or Severe Obese	28.9%	35.4%		0.139	0.079
Others	50.8%	47.3%		0.070	0.062
Comorbid Conditions					
Prior Stroke	29.1%	25.1%	< 0.001	0.089	0.051
Prior major bleeding	27.6%	28.0%	0.6	0.011	0.026
Diabetes	55.9%	57.2%	0.2	0.027	0.021
Prior AMI	10.4%	6.7%	< 0.001	0.132	0.119
Liver Disease	3.2%	3.5%	0.4	0.019	0.017
Heart Failure	41.2%	31.6%	< 0.001	0.201	0.151
Hypertension	96.8%	95.3%	< 0.001	0.076	0.030
Cardiomyopathy	50.4%	44.6%	< 0.001	0.115	0.100
Pulmonary Circulatory Dis	17.2%	14.2%	< 0.001	0.082	0.021
COPD	38.5%	37.0%	0.17	0.031	0.054
Prior Blood Transfusion	5.8%	3.5%	< 0.001	0.107	0.083
Revascularization	17.7%	15.4%	0.007	0.061	0.079
Implantable Cardiac Device	18.3%	14.8%	< 0.001	0.094	0.078
Valve Disease	42.2%	38.1%	< 0.001	0.084	0.064
Concurrent Drugs (+/− 90 days of initiating DOAC)					
SSRI/SNRI	34.0%	33.3%	0.5	0.014	0.034
Strong and moderate p-gp inhibitors	19.9%	24.5%	< 0.001	0.110	0.012
Warfarin	16.1%	19.6%	< 0.001	0.092	0.124
Pgp inducers	17.9%	18.0%	0.9	0.003	0.018
Strong p-gp and cyp3a4 dual inhibitors	26.1%	35.1%	< 0.001	0.194	0.009
P-gp and cyp3a4 inducers	17.9%	18.1%	0.9	0.003	0.017
Ace inhibitors	70.6%	69.6%	0.34	0.022	0.025
Angiotensin receptor blockers	43.9%	43.8%	0.86	0.004	0.004
Beta blockers	89.7%	89.0%	0.32	0.023	0.019
Calcium channel blockers	67.6%	64.4%	=0.003	0.067	0.026
Digoxin	23.0%	23.3%	0.7	0.008	0.003
Proton pump inhibitors	59.6%	59.2%	0.7	0.008	0.026
NSAIDS	52.6%	53.3%	0.5	0.015	0.022
Antiplatelets	31.8%	28.4%	0.001	0.074	0.067
Insulin	18.0%	17.1%	0.27	0.025	0.087
Statins	81.8%	81.6%	0.79	0.006	0.024
Renal Disease					
None or mild	3.6%	17.1%	< 0.0001	0.452	0.058
Moderate (GFR 30–60 ml/min/1.73 m2)	83.3%	77.4%		0.149	0.039
Severe (GFR < 30 ml/min/1.73 m2)	13.1%	5.5%		0.262	0.002

Abbreviations: *ACE* Angiotensin converting enzyme, *AMI* Acute myocardial infarction, *COPD* Chronic obstructive pulmonary disease, *ICD* International classification of diseases, *NSAIDS* Non-steroidal antiinflammatory drugs, *P-GP* P-glycoprotein, *CYP* Cytochrome

**Table 6:** Bivariable associations between standard dose rivaroxaban eligible patients’ characteristics on low or standard dose of rivaroxaban

	Standard dose eligible that received low dose Rivaroxaban (*n* = 2551)	Standard dose eligible that received standard dose Rivaroxaban (*n* = 9341)	*P* value	Before Matching Standardized Difference	After Matching (*N* = 2397 vs 2397) Standardized Difference
Year			*0.06*		
2010–2012	8.2%	7.1%		0.0439	0.0031
2013	17.9%	17.1%		0.0204	0.0098
2014	23.8%	22.7%		0.0256	0.0248
2015	21.4%	22.7%		0.0312	0.0051
2016–2017	28.7%	30.4%		0.0379	0.0284
Region			*0.12*		
Midwest	15.1%	14.4%		0.0178	0.1713
Northeast	1.1%	1.3%		0.0229	0.1515
South	73.1%	72.0%		0.0254	0.2038
West	10.8%	12.3%		0.0476	0.0186
Age Category			*< 0.0001*		
65–69	8.5%	19.7%		0.3266	0
70 to 74	15.9%	30.3%		0.3453	0
75 to 79	21.6%	25.5%		0.0916	0
80 to 84	25.3%	15.9%		0.2337	0
85 to 89	18.8%	6.9%		0.3622	0
90 or over	9.8%	1.7%		0.3544	0
Sex			*< 0.0001*		
Female	58.7%	43.7%		0.3044	0
Male	41.3%	56.3%			
Race Category			*< 0.0001*		
White	86.2%	86.8%		0.0164	0
Black	7.3%	6.9%		0.0144	0
Hispanic	2.4%	1.3%		0.0813	0
Asian	1.1%	0.8%		0.0304	0
Other	3.0%	4.2%		0.065	0
Smoker	21.1%	23.0%	0.05	0.044	0.003
Weight Category (based on ICD-9/ICD-10 codes for BMI)			*< 0.0001*		
Under	2.9%	1.4%		0.1038	0.0682
Healthy or Overweight	20.1%	13.6%		0.1737	0.2328
Obese or Severe Obese	22.1%	29.4%		0.1684	0.0561
Others	55.0%	55.6%		0.0126	0.2406
Comorbid Conditions					
Prior Stroke	26.9%	21.3%	< 0.001	0.1295	0
Prior major bleeding	25.2%	22.2%	0.001	0.071	0
Diabetes	43.4%	43.8%	0.76	0.0069	0.1063
Prior AMI	7.0%	5.3%	0.001	0.0701	0
Liver Disease	2.4%	2.9%	0.14	0.0337	0.0139
Heart Failure	27.0%	21.3%	< 0.001	0.1339	0
Hypertension	91.2%	90.8%	0.57	0.0128	0.0474
Cardiomyopathy	43.5%	38.3%	< 0.001	0.1063	0.1134
Pulmonary Circulatory dis	13.9%	9.8%	< 0.001	0.1277	0.0414
COPD	33.0%	29.1%	< 0.001	0.0846	0.0972
Prior blood transfusion	3.7%	1.8%	< 0.001	0.1173	0.0855
Revascularization	14.7%	13.0%	0.02	0.0495	0.08
Implantable Devices	14.1%	11.6%	< 0.001	0.0766	0.0553
Valve Disease	38.1%	35.7%	0.02	0.051	0.0347
Concurrent Drugs (+/− 90 days of initiating DOAC)					
SSRI/SNRI	33.3%	27.4%	< 0.001	0.1289	0.0682
Strong and moderate p-gp inhibitors	15.7%	16.1%	0.67	0.0096	0
Warfarin	13.2%	14.6%	0.08	0.0391	0.1199
Pgp inducers	19.5%	15.8%	< 0.001	0.097	0
Strong p-gp and cyp3a4 dual inhibitors	17.3%	14.8%	0.002	0.069	0.0232
P-gp and cyp3a4 inducers	19.5%	15.8%	< 0.001	0.097	0
Ace inhibitors	62.4%	62.1%	0.77	0.0066	0.006
Angiotensin receptor blockers	33.8%	33.8%	0.98	0.0005	0.0222
Beta blockers	85.7%	85.4%	0.74	0.0075	0.042
Calcium channel blockers	59.6%	57.3%	0.03	0.0475	0.0179
Digoxin	23.4%	19.8%	<.001	0.0892	0.0403
Proton pump inhibitors	56.2%	49.5%	< 0.001	0.1356	0.0753
NSAIDS	53.2%	48.7%	< 0.001	0.091	0.133
Antiplatelets	27.9%	22.5%	< 0.001	0.1229	0.0785
Insulin	8.5%	8.2%	0.54	0.0136	0.0247
Statins	75.4%	75.9%	0.6	0.0114	0.0608
Renal Disease					
None or mild	100.0%	100.0%			Not available due to perfect match
Moderate (GFR 30–60 ml/min/1.73 m2)					
Severe (GFR < 30 ml/min/1.73 m2)					

Abbreviations: *ACE* Angiotensin converting enzyme, *AMI* Acute myocardial infarction, *COPD* Chronic obstructive pulmonary disease, *ICD* International classification of diseases, *NSAIDS* Non-steroidal antiinflammatory drugs, *P-GP* P-glycoprotein, *CYP* Cytochrome

Mean follow-up for patients eligible for low dose dabigatran, standard dose dabigatran, low dose rivaroxaban, and standard dose rivaroxaban were 13.9, 15.1, 10.1, and 12.3 months, respectively.

### Outcomes

#### Stroke

The absolute event rates and event rates/year for ischemic stroke in each dosing category are presented in Table 7. Before adjustment for patient characteristics or propensity-match analysis, use of low dose dabigatran among patients eligible for standard dose dabigatran did not affect ischemic stroke risk. Among those eligible for standard dose rivaroxaban but receiving low dose, no significantly different risk of ischemic stroke was found. Among patients eligible for low dose dabigatran who received standard dose, we did not identify any relationship to ischemic stroke risk (Table 7). Also, among patients eligible for low dose rivaroxaban, use of standard dose rivaroxaban was not associated with increased risk of ischemic stroke. After propensity matching, we found no difference in risk of ischemic stroke in 732 patients eligible for low dose dabigatran who received low dose compared to 732 matched patients eligible for low dose dabigatran who received standard dose, or among propensity-matched patients eligible for standard dose dabigatran who received either standard dose (*n* = 1960) or low dose (*n* = 1960). Similarly, analysis of propensity matched samples of patients eligible for low dose rivaroxaban (5328 on low dose and 5328 on standard dose) or patients eligible for standard dose rivaroxaban (4500 on standard dose and 4500 on low dose) found no significant association of dose to risk of ischemic stroke (Table 7).

**Table 7:** Hazard ratios (95% Confidence Intervals) of Outcomes in Matched Cohorts of Dabigatran and Rivaroxaban low and standard doses in Non-Valvular Atrial Fibrillation

	Patients Eligible for low dose but taking standard dose vs. those taking low dose			Patients Eligible for Standard dose but taking low dose vs. those taking dose standard dose		
	Received Low Dose *Events (Events/year)*	Received Standard Dose *Events (Events/year)*	95% Confidence Interval, *p*-value	Received Low Dose *Events (Events/year)*	Received Standard Dose *Events (Events/year)*	95% Confidence Interval, *p*-value
Ischemic Stroke						
Dabigatran						
*Unadjusted*	25 (0.059)	57 (0.043)	0.77 (0.47–1.26; *p* = 0.3)	59 (0.051)	319 (0.042)	1.2 (0.92–1.6, *P* = 0.17)
*Propensity Matched*	22 (0.059)	28 (0.061)	1.13 (0.63–2; *p* = 0.69)	55 (0.049)	88 (0.071)	0.74 (0.53–1.04; *p* = 0.08)
Rivaroxaban						
*Unadjusted*	176 (0.057)	161 (0.043)	0.87 (0.7–1.1; *p* = 0.2)	89 (0.040)	256 (0.026)	*1.36 (1.07–1.7, P = 0.01)*
*Propensity Matched*	103 (0.048)	128 (0.053)	1.24 (0.96–1.6; *p* = 0.1)	78 (0.040)	73 (0.030)	1.13 (0.83–1.56, *P* = 0.43)
Major Bleeding						
Dabigatran						
*Unadjusted*	62 (0.1456)	124 (0.093)	*0.69 (0.51–0.94; p = 0.02)*	82 (0.071)	393 (0.051)	*1.35 (1.06–1.7, p = 0.01)*
*Propensity Matched*	46 (0.1228)	58 (0.1258)	1.07 (0.7–1.58 *P* = 0.75)	78 (0.069)	92 (0.075)	1.01 (0.74–1.37, *P* = 0.97)
Rivaroxaban						
*Unadjusted*	307 (0.099)	294 (0.079)	0.95 (0.8–1.1, *P* = 0.5)	147 (0.067)	426 (0.043)	*1.35 (1.12–1.6, P = 0.001)*
*Propensity Matched*	197 (0.091)	216 (0.089)	1.12 (0.92–1.35, *P* = 0.26)	131 (0.068)	116 (0.048)	1.2 (0.93–1.53, *P* = 0.21)
GI Hemorrhage						
Dabigatran						
*Unadjusted*	46 (0.108)	100(0.075)	0.75 (0.5–1.07, *P* = 0.1)	67 (0.058)	292 (0.038)	*1.47 (1.13–1.9, P = 0005)*
*Propensity Matched*	33 (0.088)	45 (0.098)	1.16 (0.74–1.82, *P* = 0.53)	63 (0.056)	68 (0.055)	1.08 (0.77–1.53, *P* = 0.66)
Rivaroxaban						
*Unadjusted*	243 (0.078)	227 (0.061)	0.93 (0.78–1.1, *P* = 0.4)	111 (0.050)	341 (0.034)	*1.28 (1.04–1.6, P = 0.02)*
*Propensity Matched*	158 (0.073)	166 (0.069)	1.07 (0.86–1.32, *P* = 0.5)	97 (0.050)	96 (0.040)	1.08 (0.82–1.43, *P* = 0.58)
Intracranial Hemorrhage						
Dabigatran						
*Unadjusted*	< 11	< 11	*0.33 (0.12–0.9; p = 0.03)*	< 11	52 (0.007)	0.77 (0.33–1.8; *p* = 0.54)
*Propensity Matched*	< 11	< 11	0.63 (0.2–1.99; *p* = 0.43)	< 11	12(0.009)	0.58 (0.22–1.54; *p* = 0.27)
Rivaroxaban						
*Unadjusted*	25 (0.008)	26 (0.007)	0.99 (0.57–1.7; *p* = 0.96)	13 (0.006)	38 (0.004)	1.33 (0.7–2.5; *p* = 0.37)
*Propensity Matched*	17 (0.008)	22 (0.009)	1.26 (0.67–2.38; *p* = 0.47)	12 (0.006)	< 11	1.57 (0.64–3.84; *p* = 0.32)

#### Major bleeding

The absolute event rates and event rates/year for bleeding complications in each dosing category are presented in Table 7. Among patients on dabigatran who met criteria for standard dose, use of low dose was associated with significantly higher risk of any major bleeding (HR = 1.44; 95% CI 1.14–1.8, *P* = 0.002, Table 7), and higher risk of GI bleeding (HR 1.48, 95% CI 1,08–2, *P* = 0.016) but not intracranial bleeding compared with patients on standard doses of dabigatran. A similar pattern of increased major bleeding risk (HR 1.34, 95% CI 1.11–1.6, *P* = 0.002) was identified among patients on rivaroxaban who met criteria for standard dose but received low dose, along with a trend towards increased risk of GI bleeding (HR 1.26, 95% CI 0.99–1.6, *P* = 0.06) but not intracranial bleeding.

In patients who met criteria for low dose dabigatran, there was lower risk of major bleeding (HR = 0.59; 95% CI 0.43–0.8, *P* < 0.001, Table 7) and intracranial bleeding (HR = 0.33; 95% CI 0.12–0.9, *P* = 0.03, Table 7) but not GI bleeding in patients who received standard compared to low dose dabigatran. Among patients who met criteria for low dose rivaroxaban, there was lower risk of GI bleeding (HR = 0.79; 95% CI 0.64–0.98, *P* = 0.03, Table 7) without differences in the risk of major, and intracranial bleeding. After controlling for patient characteristics in propensity-matched samples, we did not find any association of off-label use of low dose or standard dose and the risk of any bleeding events for either dabigatran or rivaroxaban.

#### Sensitivity analysis

We performed sensitivity analysis among patients with dose adjustments based on valid GFR measurements only and not based on pharmacologic interactions. The propensity matched analysis showed that standard dose dabigatran is associated with higher risk of stroke among patients eligible for low dose according to GFR (HR 2.6, 95% CI 1.03–6.7; *p* = 0.04). The analysis did not suggest any other significant differences in stroke and bleeding risks between off-label and standard dosing of dabigatran and rivaroxaban. The results are presented in Additional file 2: Table S2.

## Discussion

The findings of this retrospective cohort analysis of Medicare beneficiaries with AF treated with dabigatran or rivaroxaban between 2010 and 2016 can be summarized as follows: i) among patients on dabigatran or rivaroxaban who met criteria for low dose, the majority received standard dose; ii) among patients on dabigatran or rivaroxaban who met criteria for standard dose, less than one fourth received the low dose; iii) older age, female sex, black race, bleeding history, and heart failure were associated with receipt of lower than recommended dose for patients receiving dabigatran or rivaroxaban; iv) unadjusted analysis suggested that in patients receiving lower dose than recommended, the risk of any major bleeding was increased, likely reflecting higher baseline bleeding risk, while in patients receiving higher dose than recommended, the risk of bleeding was decreased; v) after risk adjusting using multivariable models or propensity-matching, off-label dosing of dabigatran or rivaroxaban was not associated with increased risk of stroke or bleeding compared to recommended dosing. An increased risk of ischemic stroke with standard dose dabigatran was found among patients eligible for low dose based on eGFR only.

The results of our analysis are in accordance with findings of previous studies which demonstrated that a significant part of AF population on DOACs receive an off-label dose [14, 15, 19]. An updated analysis of the ORBITA-AF II registry from 2013 until 2016, including 7925 AF patients treated with DOACs, showed that 84% received DOACs at standard dose (mainly rivaroxaban and apixaban, only 451 patients on dabigatran), which was consistent with FDA labeling in 96% of cases [ 15]. Reduced DOAC dose was prescribed to 16% of patients, which was consistent with FDA labeling in 43%. In unadjusted analysis, under-dosing was associated with higher rates of all-cause mortality and major bleeding [15]. Nevertheless, after risk- adjustment, the use of lower-than-recommended dose resulted in similar thromboembolic and bleeding risk compared to appropriately dosed DOAC use [15]. Our cohort included a larger sample size than ORBITA-AF II and focused on dabigatran and rivaroxaban as opposed to apixaban and rivaroxaban in ORBITA-AF II. Another methodological difference is the use of calculated creatinine clearance with the Cockcroft-Gault formula instead of the MDRD or the CKD-EPI equations for eGFR calculation that we applied. Although in clinical practice, eGFR by MDRD or CKD-EPI is more commonly used than calculated creatinine clearance, discordances in dabigatran and rivaroxaban doses may occur in up to 30% of elderly patients with creatinine clearance < 60 ml/min [ 19]. Despite these methodological differences both our analysis and the previous report from the ORBITA-AF II registry suggest that among patients on dabigatran or rivaroxaban who met criteria for low dose, the majority received standard dose. Moreover, unadjusted analysis suggested that in patients receiving lower dose than recommended, the risk of any major bleeding was increased. In both analyses, propensity matching did not reveal any significant differences in stroke and bleeding.

Yao et al.^19^ previously evaluated potential over- and under- dosing of DOACs based only on renal indication for dose reduction using the data for privately insured and Medicare Advantage enrollees. Like our study, Yao et al. found no significant relationship between risk of stroke or bleeding and dose in dabigatran or rivaroxaban-treated patients with renal indication for dose reduction. However, in aggregate analyses of patients taking dabigatran, rivaroxaban or apixaban with renal indication for low dose, patients had significantly higher bleeding risk if they received standard dose. Consistent with our study, Yao et al. also found no statistically significant relationship between dose reduction and risk of stroke or bleeding in the dabigatran- or rivaroxaban-treated patients who did not have a renal indication for low dose. In contrast to our study, Yao et al. evaluated renal indications for dose reductions only, and did not consider use of p-gp inhibitors or dual p-gp and CYP3A4 inhibitors in assessing dosing criteria.

In aggregate our findings demonstrate that decisions by healthcare providers about DOAC dosing may be based on patient clinical conditions not reflected in FDA dosing recommendations. Our analysis suggests that patients deemed by providers to be at higher bleeding risk may have received low dose DOACs even though FDA criteria suggest that they qualified for standard dose. It is noteworthy that our unadjusted analyses found higher risk of bleeding in patients who met criteria for standard dose rivaroxaban or dabigatran but received low dose, suggesting that the perception of higher bleeding risk by physicians was warranted, and may point to important patient characteristics not reflected in FDA criteria. Similarly, patients qualifying for low dose may have been prescribed standard doses if providers deemed their bleeding risk to be low.

DOAC-specific factors should predominantly affect dosing decisions. Renal function is the main indicator for low dose dabigatran and rivaroxaban. Nearly ~ 80% of ingested dabigatran is metabolized by the kidney, while ~ 30% of rivaroxaban is metabolized by the kidney. With chronic kidney disease, the half-lives of these medications are extended, leading to potentially high plasma concentrations of the medications and increased bleeding risk [20, 21]. DOAC dose adjustments based on renal function therefore reflect the increased bleeding risk in patients with compromised renal function. Several drug interactions also affect dosing of DOACs. Dabigatran is a substrate for P-glycoprotein. Concomitant use of dabigatran with P-gp inducers such as rifampin reduces the anticoagulant effect of dabigatran and should be avoided whereas use of dabigatran with P-gp inhibitors (eg, ketoconazole, dronedarone) in patients with renal disease may increase the anticoagulant effect, hence dose adjustment is required. Combined P-gp and CYP3A4 inhibitors (ketoconazole, protease inhibitors) increase the anticoagulant effects of rivaroxaban and should not be used concomitantly with rivaroxaban. In our analysis, we found a significant percentage of patients on medications interfering with the metabolism of dabigatran and rivaroxaban. Notably, receipt of standard dose dabigatran among patients qualifying for low dose dabigatran due to eGFR 30–60 ml/min with concomitant use of a p-gp inhibitor was particularly common, suggesting that some providers may not recognize the potential interaction. We also note, however, that our pharmacy data only permit evaluation of prescription fills, thus it is possible that some patients receiving concomitant p-gp inhibitors were instructed not to take them or switched to an alternative drug, in which case they would not meet criteria for low dose dabigatran. Other combinations that increase the risk of bleeding with DOACs are antiplatelet agents and non-steroidal anti-inflammatory drugs (NSAIDs), although no specific dosing adjustments are recommended for patients on these drugs. In our study, we found that use of prescription anti-platelet use among patients who met criteria for standard dose dabigatran or rivaroxaban was associated with a modestly higher use of low dose dabigatran and rivaroxaban. Similarly, use of low dose rivaroxaban was also more frequent among rivaroxaban patients taking NSAIDS.

### Limitations

An important limitation of this paper is our inability to measure GFR using the Cockcroft-Gault [CG] equation, which is reflected in FDA recommendations for DOAC dosage reductions. There are three commonly used equations for estimating GFR: the oldest is the CG equation, originally published in 1976, followed by the MDRD, updated MDRD, and CKD-EPI equations in 1999, 2005, and 2009, respectively [15, 16, 22]. The MDRD and CKD-EPI equations use serum creatinine in combination with age, sex, and race to estimate GFR, while CG also uses patient weight. The use of alternative equations for dosing decisions has been the topic of considerable debate. In clinical practice, physicians rarely use the CG-estimated GFR and instead rely on the MDRD or CKD-EPI equations, which are easy to calculate and often automatically reported with serum creatinine laboratory tests. Notably, the National Kidney Disease Education Program (NKDEP) previously indicated that either the MDRD or CG equation may be used for drug dosing decisions [23], while more recently, the National Institute of Diabetes and Digestive and Kidney Diseases suggested that either CKD-EPI or CG equations are appropriate for drug dosing purpose [24]. In contrast, a previous review of FDA-recommended drug dosing showed that the CG equation is historically the most common renal function equation cited in drug dosing recommendations [25]. As noted by Yao et al. [26], it is likely that FDA drug labels historically relied on CG-estimated GFR because this method was available before the MDRD or CKD-EPI equations were developed and widely adopted, and drug dosage recommendations have not caught up to standard clinical practice with respect to assessing renal function. Nevertheless, this poses inconsistencies with clinical practice. While for most patients, GFR estimated by the MDRD or CKD-EPI equations has reasonable concordance with CG-estimated GFR, for older patients and patients with significant comorbidity, malnutrition leading to decreased muscle mass, or morbid obesity, there may be less agreement and dosing of medications based on the former may not be consistent with FDA recommendations [ 27–29]. Schwartz [30] found that use of the MDRD or CKD-EPI equations rather than the CG equation for estimating GFR may fail to identify 20 to 50% of patients for whom reduced dabigatran and rivaroxaban doses are recommended. Thus, our analysis likely underestimates the number of patients who qualify for low dose rivaroxaban and dabigatran if decisions are based on the CG equation, thereby underestimating the proportion of patients who are overdosed, or overestimating the proportion of patients who are underdosed, relative to FDA criteria. However, they do likely reflect dosing decisions based on GFR estimates typically used in clinical practice.

Other potential limitations of this study should also be considered. First, due to the observational nature of the study, it is possible that unmeasured confounders could have affected our results in spite of using propensity matched analysis. Second, our analysis included patients over the age of 65 and the findings require validation in younger patients. Finally, we lacked detailed evidence on AF burden and estimation of thromboembolic risk. Strengths our study are the large sample size, the availability of laboratory results (GFR) for most patients, incorporating concomitant medication use for assessing dosing criteria, and application of risk adjustment methods including propensity-matched analyses.

## Conclusion

The purpose of our study was to improve understanding of safety and efficacy of DOACs in AF patients receiving low or standard dosing of these medications that was inconsistent with FDA criteria. The majority of patients qualifying for low dose DOACs received standard doses and a percentage of patients qualifying for standard dosing received low dose. After adjustment for comorbidities the risk of stroke and major bleeding was not affected by use of dose inconsistent with FDA criteria. Further validation of our results is warranted especially in patients at high thromboembolic or bleeding risk.

## Supplementary information

**Additional file 1: Table S1**. ICD 9 and 10 codes of comorbidities and outcomes included in the analysis and list of medications used for dose adjustments of dabigatran and rivaroxaban. Description of Data: All ICD 9 and 10 codes included in the analysis.

**Additional file 2: Table S2**. Hazard ratios (95% Confidence Intervals) of Outcomes in Cohorts of Dabigatran and Rivaroxaban low and standard doses in Non-Valvular Atrial Fibrillation. Description of Data: Hazard ratios of Outcomes from the Analysis of dose adjustments based on renal function only.

## Data Availability

The data that support the findings of this study are available from PearlDiver, Inc. but restrictions apply to the availability of these data, which were used under license for the current study, and so are not publicly available. Data are however available from the authors upon reasonable request and with permission of PearlDiver, Inc. (info@pearldiverinc.com).

## Abbreviations

ACE: Angiotensin-converting-enzyme
AF: Atrial fibrillation
CI: Confidence intervals
CKD: Chronic kidney disease
CrCL: Creatinine clearance)
DOACs: Direct oral anticoagulants
eGFR: Estimated glomerular filtration rate
FDA: Food and Drug Administration
ICD-9-CM: International classification of diseases–ninth revision–clinical modification
ICH: Intracranial hemorrhage
MDRD: Modification of diet in renal disease
NKDEP: National Kidney Disease Education Program
P-gp: P-glycoprotein
P-gp-Cyp3A4: P-gp and cytochrome-3A4
RCT: Randomized controlled trials
RR: Relative rates
VKA: Vitamin K antagonist
