# Supplementary material for: Characteristics and outcomes in patients with atrial fibrillation receiving direct oral anticoagulants in off-label doses
Source: BMC Cardiovasc Disord. 2020 Feb 3;20:42. doi: 10.1186/s12872-020-01340-4 (PMC6998084; doi:10.1186/s12872-020-01340-4)
Supplement: Supplementary file 1 — Additional file 1: Table S1. ICD 9 and 10 codes of comorbidities and outcomes included in the analysis and list of medications used for dose adjustments of dabigatran and rivaroxaban. Description of Data: All ICD 9 and 10 codes included in the analysis. [file 12872_2020_1340_MOESM1_ESM.docx]

**Supplemental Table 1.** ICD 9 and 10 codes of comorbidities and outcomes included in the analysis and list of medications used for dose adjustments of dabigatran and rivaroxaban.

| **Comorbid Condition** | **ICD9 Codes** | **ICD 10 Codes** | |
| --- | --- | --- | --- |
| Valve Disease | 09320-09324, 3940 -3971 , 3979 , 4240 -42499, 7463 -7466 , V422 , V433 | A5203, I050- I052, I058, I059, I060- I062, I068, I069, I070- I072, I078, I079, I080- I083, I088, I089, I091, I0989, I340- I342, I348, I349, I350-I352, I358- I362, I368- I372, I378, I379, I38, I39, Q230- Q233, Z952- Z954 | |
| Chronic Peptic Ulcer | 53141, 53151, 53161, 53170, 53171, 53191, 53241, 53251, 53261, 53270, 53271, 53291, 53341, 53351, 53361, 53370, 53371, 53391, 53441, 53451, 53461, 53470, 53471, 53491 | K254, K255, K256, K257, K259, K264, K265, K266, K267, K269, K274, K275, K276, K277, K279, K284, K285, K286, K287, K289 | |
| Renal Disease | 5853-5856 , 5859, 586 , V420 , V451 , V560 -V5632, V568 , V4511-V4512 | N183, N184, N185, N186, N189, N19, Z4901, Z4902, Z4931, Z4932, Z9115, Z940, Z992 | |
| Pulmonary Circulatory Disorder | 41511-41519, 4160 -4169 , 4179 | I2601, I2602, I2609, I2690, I2692, I2699, I270, I271, I2781, I2782, I2783, I2789, I279, I289, T800XXA, T82817A, T82818A | |
| Psychosis | 29500-2989 , 29910, 29911 | F200-F203, F205, F2081, F2089, F209, F22-F24, F250, F251, F258, F259, F28-F29, F3010-F3013, F302-F304, F308-F310, F3110-F3113, F312, F3130-F3132, F314, F315, F3160-F3164, F3170-F3178, F3181, F3189, F319, F324, F325, F3340-F3342, F348, F3481, F3489, F349, F39, F4489, F843 | |
| Peripheral Vascular Disease | 4400 -4409 , 44100-4419 , 4420 -4429 , 4431 -4439 , 44421-44422, 4471, 449, 5571 , 5579 , V434 | I700, I701, I702*, I703*, I704*, I705*, I706*, I707*, I708, I7090, I7091, I7092, I7100, I7101, I7102, I7103, I711-I729, I731, I7381, I7389, I739, I742- I744, I76, I771, I7770-I7779, I790, I791, I798, K551, K558, K559, Z95820, Z95828 | |
| Heart Failure | 39891,4280 -4289 | I0981, I501, I5020, I5021, I5022, I5023, I5030, I5031, I5032, I5033, I5040, I5041, I5042, I5043, I50810, I50811, I50812, I50813, I50814, I5082, I5083, I5084, I5089, I509 | |
| Hypertension – Uncomplicated | 4011, 4019, 64200-64204 | I10, O10011, O10012, O10013, O10019, O1002, O1003, O10911, O10912, O10913, O10919, O1092, O1093 | |
| Hypertension - Complicated | 4010, 4372 | I160, I161, I169, I674 | |
| Chronic Pulmonary Disease | 490-4928, 49300-49392,  494 -4941, 4950-505,5064 | J40, J410, J411, J418, J42, J430-J432, J438, J439, J440, J441, J449, J4520-J4522, J4530-J4532, J4540-J4542, J4550-J4552, J45901, J45902, J45909, J45990, J45991, J45998, J470, J471, J479, J60, J61, J620, J628, J630-J636, J64, J660-J662, J668,  J670-J679, J684 | |
| Diabetes without complications | 25000-25033, 64800-64804, 24900-24931 | E0800, E0801, E0810, E0811, E089, E0900, E0901, E0910, E0911, E099, E1010, E1011, E109, E1100, E1101, E1110, E1111, E119, E1300, E1301, E1310, E1311, E139, O24011, O24012, O24013, O24019, O2402, O2403, O24111, O24112, O24113, O24119, O2412, O2413, O24311, O24312, O24313, O24319, O2432, O2433, O24811, O24812, O24813, O24819, O2482, O2483, O24911, O24912, O24913, O24919, O2492, O2493 | |
| Diabetes with complications | 25040-25093, 7751, 24940-24991 | E082*, E0831*, E0832, E0833*, E0834*, E0835*, E0836, E0837*, E0839, E084*, E085*, E086*, E088*, E089*, E092*, E093*, E094*, E095*, E096*, E098, E102*, E103*, E104*, E105*, E106*, E108, E112*, E113*, E114*, E115*, E116*, E118, E132*, E133*, E134*, E135*, E136, E138, P702 | |
| Hypothyroidism | 243 -2442, 2448, 2449 | E000-E002, E009, E018, E02, E030-E033, E038, E039, E890 | |
| Liver Disease | 07022, 07023, 07032, 07033, 07044, 07054, 4560, 4561, 45620, 45621, 5710, 5712, 5713, 57140-57149, 5715, 5716, 5718, 5719, 5723, 5728, 5735, V427 | B180-B182, I8500, I8501, I8510, I8511, K700, K702, K7030, K7031, K7040, K7041, K709, K7210, K7211, K7290, K7291, K730-K732, K738-K745, K7460, K7469, K754, K7581, K760, K766, K7689, K769, Z944 | |
| Coagulation Disorder | 2860 -2869,2871, 2873 -2875, 64930-64934, 28984 | D65-D67, D680-D682, D68311, D68312, D68318, D6832, D684, D688, D689, D691, D693, D6941, D6942, D6949, D6951, D6959, D696, D7582, O99111-O99113, O99119, O9912, O9913 | |
| Obesity | 2780, 27800, 27801, 27803, 64910-64914, V8530-V8539, V8541-V8545, V8554, 79391 | E6601, E6609, E661, E662, E668, E669, O99210, O99211, O99212, O99213, O99214, O99215, R939, Z6830, Z6831, Z6832, Z6833, Z6834, Z6835, Z6836, Z6837, Z6838, Z6839, Z6841, Z6842, Z6843, Z6844, Z6845, Z6854 | |
| Fluid and Electrolyte disorder | 2760 -2769 | E860, E861, E869, E870-E876, E8770, E8771, E8779, E878 | |
| Blood Loss Anemia | 2800, 64820-64824 | D500, O9081, O99011-O99013, O99019, O9902, O9903 | |
| Anemia Deficiency | 2801 -2819, 28521-28529, 2859 | D501, D508-D513, D518-D521, D528-D532, D538, D539, D630, D631, D638, D649 | |
| Alcohol Abuse | 2910 -2913, 2915, 2918, 29181, 29182, 29189, 2919, 30300-30393, 30500-30503 | F1010, F1011, F10120, F10121, F10129, F1014, F10150, F10151, F10159, F10180-F10182, F10188, F1019, F1020, F1021, F10220, F10221, F10229, F10230-F10232, F10239, F1024, F10250, F10251, F10259, F1026, F1027, F10280-F10282, F10288, F1029, F10921, F1094, F10950, F10951, F10959, F1096, F1097, F10980-F10982, F10988, F1099 | |
| Drug Abuse | 2920, 29282-29289, 2929, 30400-30493, 30520-30593, 64830-64834 | F111*, F112* , F121*, F122*, F131*, F132*, F141*, F142*, F151*, F152*, F161*, F162* , F181*, F182*, F191*, F192*, F550-F554, F558, O99320-O99325 | |
| Depression | 3004 , 30112, 3090, 3091, 311 | F320-F322, F323, F328, F3281, F3289, F329-F333, F338, F339, F341, F4321 | |
| **Additional comorbidities relevant to atrial fibrillation** | | | |
| Atrial fibrillation | 42731 | | I480*, I481*, I482*, I489* |
| Sleep Apnea | 78057, 32723 | | G4730,G4733 |
| Prior Acute Myocardial Infarction | 410.* | | I21.*, I22.* |
| Prior Coronary Artery Disease | 410.*, 411.*, 412.*, 414.* | | I21*, I22*, I24*, I25* |
| Pulmonary Embolism / Deep Vein thrombosis | 4151-4159, 4532-4534, 45382, 45384, 45385, 45386 | | I26* |
| Prior pacemaker | V4501 | | Z950, Z4501 |
| Prior Implantable Defibrillator | V4502 | | Z95810, Z4502 |
| Prior Dementia | 290.*, 2941, 2942, 3312 | | G311, F02* - F03* |
| Prior revascularization | V4581, V4582 | | Z951,Z9861 |
| Tobacco Use, Smoking | 3051, V1582 | | F17200, Z89891 |
|  | ICD-9 code | | ICD-10 code |
| **Clinical outcomes** | | | |
| Ischemic stroke | 433*, 434*, 436*, 4371, 4378, 4379 | | I63, I65, I66, I6781, I6782, I6789, I679 |
| GI hemorrhage | 4552, 4555, 4558,  4560, 45620, 5307, 53082, 5310-5316, 5320-5326, 5330-5336, 5340-5346, 53501-53561, 56202-56203, 56212-56213, 5693, 56985, 5780, 5781, 5789, 53783, 56881 | | I8501, I8511, K644*, K648*  K226*, K228*, K250*-K256*, K260*-K266*, K270*-K276*, K280*-K286*, K2901, K2911, K2921, K2931, K2941, K2951, K2961, K2971, K2981, K2991  K5701, K5711, K5713, K5721, K5731, K5733, K5741, K5751, K5753, K5781, K5791, K5793  K625, K5521, K920*, K921*, K922*, K31811, K661* |
| Cerebral hemorrhage | 430*, 431*, 432* | | I60*, I61*, I62* |
| Other major bleeding | 59970, 59971, 59972, 71911, 7847*, 7848x, 7863*, 4230*, 4590*, 852*, 853* | | R310*, R311*, R312*, R319*, M25019, R040*, R041*, R042*, R0481, R0489, R049, I312*, R58*  S064X0A, S064X1A, S064X2A, S064X3A, S064X4A, S064X5A, S064X6A, S064X7A, S064X8A, S064X9A, S065X0A, S065X1A, S065X2A, S065X3A, S065X4A, S065X5A, S065X6A, S065X7A, S065X8A, S065X9A, S066X0A, S066X1A, S066X2A, S066X3A, S066X4A, S066X5A, S066X6A, S066X7A, S066X8A, S066X9A. |
| **Medications used for dose adjustments** | | | |
| P-glycoprotein inhibitors (used for dabigatran dose adjustment): dronedarone, cyclosporine, itraconazole, tacrolimus, ketoconazole | | | |
| Dual P-gp-Cyp3A4 inhibitor (used for rivaroxaban dose adjustment): ketoconazole, fluconazole, itraconazole, cobicistat, conivaptan, indinavir, voriconazole, posaconazole, nefazodone HCL, ritonavir, saquinavir, telithromycin | | | |
