# Supplementary material for: Characteristics and outcomes in patients with atrial fibrillation receiving direct oral anticoagulants in off-label doses
Source: BMC Cardiovasc Disord. 2020 Feb 3;20:42. doi: 10.1186/s12872-020-01340-4 (PMC6998084; doi:10.1186/s12872-020-01340-4)
Supplement: Supplementary file 2 — Additional file 2: Table S2. Hazard ratios (95% Confidence Intervals) of Outcomes in Cohorts of Dabigatran and Rivaroxaban low and standard doses in Non-Valvular Atrial Fibrillation. Description of Data: Hazard ratios of Outcomes from the Analysis of dose adjustments based on renal function only. [file 12872_2020_1340_MOESM2_ESM.docx]

**Supplemental Table 2.** Hazard ratios (95% Confidence Intervals) of Outcomes in Cohorts of Dabigatran and Rivaroxaban low and standard doses in Non-Valvular Atrial Fibrillation.

|  | **Patients Eligible for low dose but taking standard dose vs. those taking low dose** | | | **Patients Eligible for Standard dose** **but taking low dose vs. those taking dose standard dose** | | |
| --- | --- | --- | --- | --- | --- | --- |
|  | **Received Low Dose**  *Events (Events/year)* | **Received Standard Dose**  *Events (Events/year)* | **Hazard Ratio, 95% Confidence Interval,**  **p-value** | **Received Low Dose**  *Events (Events/year)* | **Received Standard Dose**  *Events (Events/year)* | **Hazard Ratio, 95% Confidence Interval,**  **p-value** |
| **Ischemic Stroke** |  |  |  |  |  |  |
| **Dabigatran** |  |  |  |  |  |  |
| *Unadjusted* | 11 (0.058) | 27 (0.098) | 2.11 (1.04-4.25, P=0.03) | 73 (0.052) | 335 (0.038) | 1.30 (1.01-1.67, P=0.04) |
| *Propensity Matched* | 6 (0.038) | 16 (0.083) | 2.63 (1.03-6.72, P=0.04) | 72 (0.052) | 81 (0.0529) | 0.96 (0.70-1.32, P=0.81) |
|  |  |  |  |  |  |  |
| **Rivaroxaban** |  |  |  |  |  |  |
| *Unadjusted* | 175 (0.631) | 153 (0.298) | 0.99 (0.79-1.23; P=0.90) | 99 (0.253) | 278 (0.315) | 1.41 (1.12-1.78, P=.003) |
| *Propensity Matched* | 106 (0.509) | 118 (0.294) | 1.11 (0.85-1.44; P=0.46) | 83 (0.234) | 84 (0.297) | 1.03 (0.76-1.40; P=0.84) |
|  |  |  |  |  |  |  |
| **Major Bleeding** |  |  |  |  |  |  |
| **Dabigatran** |  |  |  |  |  |  |
| *Unadjusted* | 43 (0.226) | 38 (0.140) | 0.71 (0.46-1.11, P=0.14) | 100 (0.072) | 463 (0.053) | 1.30 (1.04-1.61, p=0.02) |
| *Propensity Matched* | 28 (0.179) | 26 (0.135) | 0.86 (0.50-1.47 P=0.60) | 98 (0.071) | 105 (0.069) | 1.02 (0.77-1.34, P=0.91) |
|  |  |  |  |  |  |  |
| **Rivaroxaban** |  |  |  |  |  |  |
| *Unadjusted* | 296 (1.067) | 255 (0.498) | 0.98 (0.83-1.16, P=0.90) | 161 (0.411) | 475 (0.539) | 1.33 (1.12-1.60, P=0.002) |
| *Propensity Matched* | 190 (0.912) | 198 (0.494) | 1.03 (0.85-1.26, P=0.76) | 140 (0.395) | 125 (0.441) | 1.16 (0.91-1.48, P=0.22) |
|  |  |  |  |  |  |  |
| **GI Hemorrhage** |  |  |  |  |  |  |
| **Dabigatran** |  |  |  |  |  |  |
| *Unadjusted* | 34 (0.179) | 29(0.105) | 0.69 (0.42-1.14, P=0.15) | 78 (0.056) | 352 (0.098) | 1.33 (1.04-1.70, P=0.02) |
| *Propensity Matched* | 23 (0.147) | 23 (0.119) | 0.92 (0.52-1.64, P=0.80) | 77 (0.056) | 81 (0.083) | 1.03 (0.76-1.41, P=0.84) |
|  |  |  |  |  |  |  |
| **Rivaroxaban** |  |  |  |  |  |  |
| *Unadjusted* | 236 (0.851) | 201 (0.392) | 0.97 (0.80-1.17, P=0.74) | 121 (0.309) | 375 (0.426) | 1.27 (1.03-1.56, P=0.02) |
| *Propensity Matched* | 157 (0.754) | 159 (0.396) | 1.01 (0.80-1.25, P=0.98) | 108 (0.305) | 97 (0.342) | 1.16 (0.88-1.52, P=0.30) |

|  |  |  |  |  |  |  |
| --- | --- | --- | --- | --- | --- | --- |
| **Intracranial Hemorrhage** |  |  |  |  |  |  |
| **Dabigatran** |  |  |  |  |  |  |
| *Unadjusted* | <11 | <11 | 0.41 (0.08-2.27, P=0.31) | <11 | 55 (0.006) | 1.09 (0.55-2.13, P=0.81) |
| *Propensity Matched* | <11 | <11 | Undefined | <11 | 12(0.008) | 0.80 (0.34-1.91, P=0.62) |
|  |  |  |  |  |  |  |
| **Rivaroxaban** |  |  |  |  |  |  |
| *Unadjusted* | 25 (0.090) | 23 (0.045) | 1.03 (0.59-1.82, P=0.92) | 13 (0.033) | 41(0.047) | 1.26 (0.67-2.34, P=0.47) |
| *Propensity Matched* | 14 (0.067) | 17 (0.042) | 1.18 (0.58-2.40, P=0.64) | <11 | 12(0.042) | 0.87 (0.37-2.01, P=0.74) |
